# A psychometric analysis of the adapted historical loss scale and historical loss associated symptoms scale among native Hawaiian adults

**DOI:** 10.3389/fpubh.2024.1356627

**Published:** 2024-07-12

**Authors:** Mapuana C. K. Antonio, Samantha Keaulana, Claire Townsend Ing, Madison Williams, Adrienne Dillard, Bridget Puni Kekauoha, Meghan Kenney, Sarah Momilani Marshall, Kevin Cassel, Scott Abrigo, Michelle Kauhane, Joseph Keawe‘aimoku Kaholokula

**Affiliations:** ^1^Native Hawaiian and Indigenous Health, Office of Public Health Studies, Thompson School of Social Work & Public Health, University of Hawai‘i at Mānoa, Honolulu, HI, United States; ^2^Department of Native Hawaiian Health, John A. Burns School of Medicine, University of Hawai‘i at Mānoa, Honolulu, HI, United States; ^3^Kula no na Po‘e Hawai‘i, Honolulu, HI, United States; ^4^Department of Social Work, Thompson School of Social Work & Public Health, University of Hawai‘i at Mānoa, Honolulu, HI, United States; ^5^University of Hawai‘i Cancer Center, University of Hawai‘i at Mānoa, Honolulu, HI, United States; ^6^Kapolei Community Development Corporation, Kapolei, HI, United States

**Keywords:** historical trauma, intergenerational trauma, health and healing, indigenous health, psychometrics, factor analysis

## Abstract

**Objectives:**

The Historical Loss Scale (HLS) and Historical Loss Associated Symptoms Scale (HLASS) are standardized measures that have been accepted and previously validated among North American Indigenous communities and allow researchers to measure the impact of Historical Loss. Evidence of the psychometric properties of this instrument have not been assessed for Native Hawaiians, the Indigenous peoples of Hawai‘i. The purpose of this study is to investigate the psychometric properties of the adapted HLS (aHLS) and HLASS for adults from multiple Hawaiian Homestead Communities throughout Hawai‘i.

**Methods:**

Data are based on cross-sectional surveys administered between 2014 and 2020. The final sample included 491 Native Hawaiian adults who were predominantly female (67.3%) and between the ages of 18–90 years, who were part of the larger study entitled the Hawaiian Homestead Health Survey. Factor analyses were conducted to determine the final model structures of each scale. Reliability and correlation matrices of items are also reported.

**Results:**

The final factor structure of the aHLS model suggested 3 factors: (1) General loss of culture or cultural loss, (2) Intergenerational loss, and (3) Distrust and destruction of traditional foods. The final HLASS model also suggested 3 factors: (1) Depression and Anger, (2) Shame and Anxiety, and (3) Re-experiencing, fear, and avoidance.

**Conclusion:**

These findings have implications for future research, practice, and education that explores the role of Historical Loss and associated symptoms in Native Hawaiians and Indigenous communities at large. In particular, measuring historical loss and associated symptoms in Hawaiian Homestead communities paves the way for quantitative assessments of historical trauma and healing in these communities.

## 1 Introduction

Native Hawaiians, the Indigenous peoples of Hawaiʻi, were once described by foreign and western visitors and settlers as being robust and capable of great physical activity (1). Native Hawaiian lifestyles and worldviews center relationships where health is viewed as a sacred force maintained through balance and holistic approaches to health. The physical and environmental manifestation of balance was often reflected through complex, agricultural systems such as ahupua‘a and ‘aha moku systems. Unbeknownst to foreigners, Native Hawaiians possessed an interdependent relationship with ʻĀina (land, nature, environment, that which feeds) that manifested in cultural practices and beliefs, which helped them maintain their physical, mental, spiritual, and emotional health (2–5). The large influx of westerners and foreigners in the Hawaiian Kingdom in the 1800s influenced many policies that interrupted the Native Hawaiian way of life, which shifted the sociopolitical power, contributing to land displacement and privatization of land, ultimately impacting Native Hawaiian health and wellbeing (6–10). After the illegal overthrow of the Hawaiian Kingdom in 1893, Hawaiʻi quickly became a major U.S. military site and tourist attraction. Since western contact, Native Hawaiians continue to face inequitable opportunities, including lower socioeconomic status and health inequities that negatively impact mental, physical, spiritual, and emotional health (11–13).

Today, the biomedical health status of Native Hawaiians remains one of the poorest in the state when compared to other major ethnic groups. Native Hawaiians make up 22.7% of Hawaiʻi’s population (14) and are projected to grow to 47% by 2025 (15), yet they experience one of the lowest life expectancies in Hawaiʻi (16, 17). Chronic conditions, such as cardiovascular diseases, obesity, and diabetes are more prevalent in Native Hawaiians compared to other major ethnic groups (16, 18). Additionally, Native Hawaiians experience morbidity prevalence and mortality of the top five leading causes of death at greater rates than other major ethnic groups (16, 18).

Indigenous researchers theorize the health status of Indigenous populations as a direct result of historical trauma (19–22). Historical trauma refers to the enduring impact of systemic oppression, violence, dispossession, or other forms of harm on a community and collective group of people (19). Both current and past experiences contribute to collective trauma responses. The effects of these events and experiences can be transmitted intergenerationally, with long-lasting impacts that influence the biological, social, emotional, and mental health and wellbeing of the affected community (19). The Historical Trauma Conceptual Model conceptualizes the way mass trauma experiences, like loss of land and physical/psychological violence, impacts primary generations and secondary/subsequent generations through unresolved trauma responses (23).

In alignment with this model, the historical loss and cultural trauma experienced by Native Hawaiians directly impact generations subjected to mass death due to infectious disease, assimilative policies including language bans, and the illegal overthrow of the Hawaiian Kingdom (2, 9, 10, 24). Therefore, a culmination of traumatic events in Hawaiʻi, like colonization, cultural degradation, depopulation, and land ownership policies, are arguably a determinant of health for contemporary Native Hawaiians (12, 25). These historical losses continue to impact Native Hawaiians directly and indirectly today. We must validly and reliably measure Historical Loss to better understand and address their impacts. Whitbeck and colleagues developed two scales, the Historical Loss Scale (HLS) and the Historical Loss Associated Symptoms Scale (HLASS) to begin to enumerate historical trauma among North American Indigenous communities, who also faced mass trauma from colonialism (22). Thus, this study explores the psychometric properties of an adapted HLS and the HLASS among Native Hawaiian communities.

### 1.1 Historical trauma as a determinant of health

Scholars theorize the way historical trauma serves as an intergenerational determinant of health (19, 20, 22, 23). For instance, historical trauma theory builds on existing theoretical frameworks including psychosocial theory, political economic theory, and social ecological systems theory. These collective frameworks theorize the way disease is linked to physical and psychological stress that stem from the larger environment, political, economic, and structural determinants of health, and are multilevel, thereby impacting individuals intergenerationally and across the lifespan (23). Thus, historical trauma, or the deliberate and systematic trauma inflicted on a collective group or community results in disparities and inequities that persists across generations.

As with measurement and survey instrumentation as a whole, standardized and empirical measures of historical trauma are important to appropriately measure the health impacts of historical trauma, particularly as a risk factor and possible mediator and moderator of health. Valid and reliable measures may also advance health practice, research, and policy by exploring these impacts on health and wellbeing. For instance, empirical evidence that demonstrates the magnitude and severity of experiences related to displacement, violence, economic and political destruction and cultural dispossession that specifically result from colonialism, ongoing cultural and historical trauma, assimilative policies, and systemic oppression among Indigenous Peoples will help to inform health practice, research, and policy that fosters self-determination, and thus, health and wellbeing at large.

While this may be the case, limitations exist among measurements of historical trauma, primarily due to the difficulty in capturing the complex experiences of historical trauma that are unique to specific communities, with the potential for these impacts to vary between and within groups of people. In 2019, Gone et al. published a systematic literature review that explored literature related to Indigenous Historical Trauma (26). As noted by the authors, findings demonstrated impressive efforts and attempts to measure the impact of Indigenous Historical Trauma, yet the body of literature does not coherently demonstrate the empirical impacts of Indigenous Historical Trauma. Despite these limitations, and although the systematic literature review focused on Indigenous populations in the United States and Canada, the authors suggest refining existing measures of Indigenous Historical Trauma as solutions to remedy the urgent and pressing need to better understand the role of Indigenous Historical Trauma in health disparities and inequities experienced by Indigenous Peoples.

### 1.2 Historical loss scale (HLS)

One of the most widely used or adapted measures of Indigenous Historical Trauma is the Historical Loss Scale (HLS). The HLS was originally developed by Whitbeck and colleagues and validated with 143 American Indian adults who were parents of children between the ages of 10–12 years (22). According to the Exploratory Factor Analysis (EFA), the HLS was proposed as a unidimensional construct consisting of 12 items, which demonstrated high internal reliability (22). The original 12 items consisted of: (1) The loss of our land, (2) The loss of our language, (3) Losing our traditional spiritual ways, (4) The loss of our family ties because of boarding schools, (5) The loss of families from the reservation to government relocation, (6) The loss of self respect from poor treatment by government officials, (7) The loss of trust in White individuals from broken treaties, (8) Losing our culture, (9) The losses from the effects of alcoholism on our people, (10) Loss of respect by our children and grandchildren for elders, (11) Loss of our people through early death, (12) Loss of respect by our children for traditional ways.

Since the original publication of the HLS, researchers have proposed a multi-dimensional Historical Loss construct. For instance, in a later publication, Whitbeck and colleagues found that the original one-factor model demonstrated poor model fit (27). As a result, researchers proposed a 10-item 2-factor model. Factor one included items on government and institutional policies and practices including loss of land, loss of family ties because of boarding schools, loss due to government relocation, broken treaties, and poor treatment by government officials. Factor two focused on personal and cultural losses, including loss of language and spiritual ways, and loss of people to early death and via the effects of alcoholism (27). In another study, Armenta and colleagues proposed a higher-order construct of Historical Loss based on an adapted 10-item scale administered to 636 North American Indigenous adolescents (20). Based on the findings, researchers proposed a three-factor model: (1) General loss of culture, (2) Loss of people, and (3) Cultural mistreatment (20). Although the model with three subfactors and a larger Historical Loss factor demonstrated poor model fit, proposed models for second-order and third-order models indicated good model fit (20).

### 1.3 Historical loss associated symptoms scale (HLASS)

The HLASS was also originally developed by Whitbeck and colleagues based on the same sample of 143 American Indian adult parents and children (22). The 17-item HLASS was developed to empirically link perceptions of historical loss to various psychological symptoms found in qualitative literature. An exploratory factor analysis yielded two factors, anxiety/depression and anger/avoidance (22). Anxiety/depression includes five items reporting the experience of depression, anxiety, loss of concentration, isolation, and loss of sleep. Anger/avoidance includes seven items, which measures anger, discomfort around white people, shame, rage, fear and distrust, feeling like traumatic events are happening again, and avoiding places. A confirmatory factor analysis supported these results and a structural equation model yielded construct loading that were also consistent with the 2-factor model (22).

Despite evidence of a multidimensional, 2-factor model, other studies have used the HLASS as a single-factor. Wiechelt and colleagues examined the relationship between historical trauma symptoms and substance use and family cohesion among 120 adult American Indians (28). Results indicated that historical trauma symptoms were associated with past month alcohol use, lifetime use of non-marijuana illicit drugs, and lower family cohesion. The authors used the 12-item scale with single factor model and reported high internal consistency (*α* = 0.89). Anastario and colleagues also examined the relationship between historical trauma symptoms and sexual risk behavior among 120 young American Indian men (29). As a single factor, the HLASS had high internal consistency (*α* = 0.88). The 2-factor model also yielded good internal consistency, with a Cronbach’s alpha of 0.73 for anxiety/depression and a Cronbach’s alpha of 0.84 for anger/avoidance. Both factors were related to an increased likelihood of respondents’ having sex with multiple concurrent partners.

### 1.4 Adapting HLS for native Hawaiians

Both HLS and HLASS were developed for and by North American Indigenous communities to assess Historical Loss and associated symptoms. In the Native Hawaiian context, the HLS was previously adapted to determine the relationship between Historical Loss and other measures of historical trauma (i.e., historical traumatic events), discrimination, and substance use (30). In the study by Pokhrel and Herzog, researchers omitted items from the original scale that were not relevant to the Native Hawaiian community, which resulted in an adapted 8-item survey administered to 128 Native Hawaiian community college students (*M*age = 27.5; SD = 9.5; 65% women). The 8-item survey administered by Pokhrel and Herzog consisted of the following items: (1) Loss of land, (2) Loss of language, (3) Loss of traditional spiritual ways, (4) Loss of self-respect from poor treatment by government officials, (5) Loss of culture, (6) Loss of respect by children for elders, (7) Loss of people through early death, and (8) Loss of respect by children for traditional ways.

Similar to Whitbeck et al.’s original scale, participants reported the frequency of thinking about historical loss as well as historical traumatic events. Historical loss and historical traumatic events both loaded on a factor of historical trauma. Interestingly, and according to structural equation modeling, the historical trauma factor had a weak and direct effect on substance use, which was mediated by perceived discrimination, resulting in a weak and indirect relationship with substance use. Despite the findings of this study, reliability and goodness of fit statistics were not reported for the HLS. Nonetheless, the adapted HLS was found to be positively and significantly correlated with historical traumatic events and discrimination, indicating convergent validity. For instance, the HLS implemented by Pokhrel and Herzog were moderately and positively associated with historical traumatic events, which was measured by eight items relevant to Native Hawaiian experiences and the perception of participants experiencing these historical traumatic events as well as the participant’s parents, grandparents, great-grandparents, and great-great-grandparents.

### 1.5 Context of study

The Hawaiian Homes Commission Act was signed by U.S. Congress in 1921, with the goal of providing “rehabilitation” for the Native Hawaiian people. These government-sponsored lands span to include 203,500 acres throughout the islands of Hawai‘i and are designated to Native Hawaiians who meet the government-assigned blood quantum qualifications, with legal implications for blood quantum as a government metric of Hawaiian identity and ability to reside on Hawaiian Homes. Despite experiences of adversity related to structural issues, Hawaiian Homestead communities serve as a kīpuka (a calm place in a high sea, deep place in a shoal, variation or change of form, or opening in a forest), or space where cultural practices and values continue to be perpetuated despite experiences related to ongoing cultural and historical trauma.

### 1.6 Purpose of study

Given the need to explore historical loss and associated symptoms in Native Hawaiian communities, the present study aimed to psychometrically test the newly adapted HLS (aHLS) and HLASS scales to support measurement of historical loss and associated symptoms among a Native Hawaiian population residing on Hawaiian Homestead communities.

## 2 Methods

### 2.1 Community-based participatory research approach

Community-based participatory research (CBPR) approaches promote social justice and health equity through research approaches that (re)distribute power to communities and ensures community and academic partners are engaged at every step of the research process (30). In this study, CBPR approaches were employed to develop a comprehensive health survey that was mailed to participants from select Hawaiian Homestead communities (See 2.2. Population/Sample Description for further description). Community and academic partnerships previously existed from other research projects and efforts to improve health outcomes for Native Hawaiian health, including a decade-long community-based research project that focused on diabetes and obesity-prevention. During the early phases of the research process, which started in 2013, community and academic partners engaged in various meetings to identify priority areas of interest for the comprehensive health survey. The aHLS and HLASS were of interest to better understand how measures of historical loss were associated with health and health-related outcomes for Native Hawaiian communities.

A comprehensive list of measures was compiled, which included the original HLS and HLASS. Community partners and leaders assisted with survey item adaptation of the HLS. When adapting items from the HLS, there was a strong interest to ensure items were appropriately measuring experiences of Native Hawaiians. Community partners and leaders dedicated time to review each HLS item and adapted the overall scale to better align with Native Hawaiian experiences and communities. Thus, there was a desire to omit items that did not appear relevant to Native Hawaiian experiences and re-word items to increase receptivity by individuals from Native Hawaiian communities. At the time of these initial meetings, there were no publications about the HLS and HLASS being assessed or measured in the Native Hawaiian community.

Given the nature of the survey funding mechanism, the comprehensive survey also included items that measured cancer prevalence, cancer-related health outcomes, and cancer-related behaviors. Based on community guidance and previous academic research, additional sections were included in the comprehensive health survey to assess for variables including demographics and socio-demographic factors; general health and health outcomes including family history of health conditions and health behaviors (including tobacco use, nutrition, physical activity); health-related factors including psychosocial factors, resilience-based factors, and social relations including discrimination, Hawaiian cultural affiliation, Historical Loss, and neighborhood connectedness. Community leaders and partners of this CBPR partnership were engaged in every step of the research process. In alignment with CBPR principles, community partners led survey recruitment, data entry, and data management. Community partners also assisted with the interpretation of data and provided annual community reports to disseminate a summary of the survey data to participants of the Hawaiian Homestead Health Survey. For instance, discussions around the findings and interpretation of data from this study was incorporated in annual meetings held by the Hawaiian Homestead Health Survey team. Furthermore, the final factor labels of study are based on the ongoing discussions and meetings that took place with the Hawaiian Homestead Health Survey team.

### 2.2 Population/sample description

The final sample of our study comprised 491 Native Hawaiian adult residents of the Hawaiian Homestead Lands from O‘ahu and Hawai‘i island. Lessees from select Hawaiian Homestead communities were invited to participate in the study based on a mailing list provided by the partner Hawaiian Homestead community. Participants were recruited using a convenience sampling method and based on established relationships with leaders and partners of the Hawaiian Homestead Health Survey team. Additionally, surveys were addressed to the specific “Lessee” of the property. To increase anonymity, the research team had no way to confirm who completed the survey. Instead, participant recruitment and compensation was provided by household.

The general response rate was around 30% of all households, with a total of 512 participants. Of the 512 respondents, 21 did not identify as Hawaiian and were excluded from the final sample of this study, for a final sample size of 491. All participants in the final sample were Native Hawaiian adults, at least 18 years of age. Ages of participants ranged from 18 to more than 90 years, with a mean age of 55.5 (SD = 14.9). Survey respondents were primarily female (*N* = 330, 67.28%) and married (*N* = 289, 42.6%). The majority of the participants had a high school diploma (35.5%), followed by some college or technical school training (31.6%). Table 1 summarizes the characteristics of participants from this study.

**Table 1:** Participant characteristics (*N* = 491).

Characteristics	Mean (SD) or *N* (%)
Age (years)	55.5 (14.9)
Female (vs. male)	330/491 (67.28%)
Marital status	
Currently single (not married)	113 (23.2%)
Currently married	289 (42.6%)
Divorced/separated/widowed	85 (17.5%)
Educational attainment	
No high school diploma	20 (4.1%)
High school graduate/general education development	173 (35.5%)
Some college/technical school	154 (31.6%)
College graduate	141 (28.9%)
Income (*n* = 476)	
0-less than $30,000	88 (18.5%)
$30,000 to less than $50,000	92 (19.3%)
$50,000 to less than $75,000	76 (16.0%)
$75,000 or more	189 (39.7%)
Do not know or unsure	31 (6.5%)

### 2.3 Measures

The primary measures of this study included the aHLS and HLASS, which were included in the Hawaiian Homestead Health Survey. The HLS was originally a 12-item scale, which measured Historical Loss as a unidimensional factor. In alignment with CBPR approaches, the research team consulted community leaders regarding the face validity of the original 12-item scale. Based on continual community feedback, the final adapted scale consisted of eight adapted items. The newly adapted scale included the following items: (1) The taking of our land, (2) Fewer and fewer people using our traditional language, (3) Destruction of our culture and traditional spiritual ways, (4) Loss of respect for elders by our children and grandchildren, (5) Loss of respect by our children for traditional ways, (6) Distrust, resentment, or fear toward white people, (7) Destruction or damage of traditional foods, and (8) The destruction of natural resources and beauty due to pollution, mining, and other industries. This led to the adapted HLS (aHLS) (Table 2).

Similar to the original HLS scale, respondents who completed the aHLS rated the frequency of thinking about Historical Loss on a 6-point Likert scale, ranging from 1 (never) to 6 (several times a day). Thus, the following scores indicated the frequency in which participants thought of the historical loss item: 1 (never), 2 (yearly or only at special times), 3 (monthly), 4 (weekly), 5 (daily), and 6 (several times a day). In the original scale, items were reverse-coded, and thus, higher scores indicated more frequency of thoughts related to Historical Loss. In the aHLS, higher scores indicate greater frequency of thinking about Historical Loss.

The HLASS is a 12-item scale which measures the frequency of experiencing specific symptoms when thinking about the Historical Loss listed in the HLS. The specific symptoms include depression, anger, anxiety, being uncomfortable around White people, shame, loss of concentration, isolation, loss of sleep, rage, being fearful and distrusting of White people, feeling like it is happening again, and avoiding places that remind them of Historical Loss (Table 3). After much discussion and taking a CBPR approach, it was determined that the original HLASS scale would be appropriate to include in the comprehensive health survey without any adaptations. In other literature, these items have been found to load on to two factors, anxiety/depression (five items) and anger/avoidance (seven items) (22). Respondents rate the frequency of experiencing each feeling on a 5-point Likert scale, from 1 (never) to 5 (always). Thus, the following scores indicated the frequency in which participants experienced the associated symptoms when thinking about the historical loss: 1 (never), 2 (seldom), 3 (sometimes), 4 (often), and 5 (always). Similar to the HLS, higher scores of HLASS items indicate greater frequency of experiencing the associated symptoms (Table 4).

**Table 2:** List of historical loss scale items.

Items from Whitbeck et al.’s scale	Items from Pokhrel & Herzog’s scale	Items from the aHLS from the Hawaiian homestead health survey
(1) The loss of our land	(1) Loss of land	(1) The taking of our land
(2) The loss of our language	(2) Loss of language	(2) Fewer and fewer people using our traditional language
(3) Losing our traditional spiritual ways	(3) Loss of traditional spiritual ways	(3) Destruction of our culture and traditional spiritual ways
(4) The loss of our family ties because of boarding schools		
(5) The loss of families from the reservation to government relocation		
(6) The loss of self respect from poor treatment by government officials	(4) Loss of self-respect from poor treatment by government officials	
(7) The loss of trust in White individuals from broken treaties		(6) Distrust, resentment, or fear toward white people
(8) Losing our culture	(5) Loss of culture	
(9) The losses from the effects of alcoholism on our people		
(10) Loss of respect by our children and grandchildren for elders	(6) Loss of respect by children for elders	(4) Loss of respect for elders by our children and grandchildren
(11) Loss of our people through early death	(7) Loss of people through early death	
(12) Loss of respect by our children for traditional ways	(8) Loss of respect by children for traditional ways	(5) Loss of respect by our children for traditional ways
		(7) Destruction or damage of traditional foods
		(8) The destruction of natural resources and beauty due to pollution, mining, and other industries

The items listed above are accompanied with the following prompt: Native Hawaiians have experienced many events, traumas, and changes since coming into contact with Europeans (White individuals). Below is a series of things like this that people have mentioned to us. Please indicate how often you think of these whether or not you are Native Hawaiian. Answer responses are scored as: (1) never, (2) yearly or only at special times, (3) monthly, (4) weekly, (5) daily, and (6) several times a day. Scoring follows the recommendation provided by Whitbeck et al. of having higher scores indicate higher levels of historical loss.

**Table 3:** List of historical loss associated symptoms scale items.

Items from Whitbeck et al.’s original scale
(1) Sadness or depression
(2) Anger
(3) Anxiety or nervousness
(4) Uncomfortable around white people (when you think of these losses)
(5) Shame (when you think of these losses)
(6) A loss of concentration
(7) Feel isolated or distant from other people (when you think of these losses)
(8) A loss of sleep
(9) Rage
(10) Fearful or distrust the intention of white people
(11) Feel like it is happening again
(12) Feel like avoiding places or people that remind you of these losses

The items listed above are accompanied with the following prompt: Now I would like to ask you about how you feel when you think about these losses. Answer responses are scored as: (1) never, (2) seldom, (3) sometimes, (4) often, and (5) always. Scoring follows the recommendation provided by Whitbeck et al. of having higher scores indicate higher levels of historical loss associated symptoms.

**Table 4:** Mean scores of the adapted historical loss scale (aHLS) and historical loss associated symptoms scale (HLASS).

aHLS or HLASS item or factor	Mean (SD)
Mean of adapted historical loss scale (aHLS) items*	
aHLS1 (the taking of land)	3.0 (1.4)
aHLS2 (fewer and fewer people using our traditional language)	3.0 (1.4)
aHLS3 (destruction of our culture and traditional spiritual ways)	3.1 (1.5)
aHLS4 (loss of respect for elders by our children and grandchildren)	3.6 (1.5)
aHLS5 (loss of respect by our children for traditional ways)	3.3 (1.5)
aHLS6 (distrust, resentment, or fear toward white people)	2.5 (1.5)
aHLS7 (destruction or damage of traditional foods)	2.7 (1.5)
aHLS8 (the destruction of natural resources and beauty due to pollution, mining, and other industries)	3.5 (1.6)
Mean of aHLS score based on Armenta’s study*	
Mean of cultural loss factor	3.0 (1.3)
Mean of cultural mistreatment factor	3.5 (1.4)
Mean of aHLS score based on EFA*	
Mean of cultural loss factor	3.0 (1.3)
Mean of intergenerational loss factor	3.5 (1.4)
Mean of distrust and destruction of traditional foods factor	2.5 (1.4)
Mean of hierarchical factor	3.0 (1.2)
Historical loss associated symptoms scale (HLASS) items**	
HLASS1 (sadness or depression)	2.5 (1.1)
HLASS2 (anger)	2.6 (1.1)
HLASS3 (anxiety or nervousness)	2.0 (1.0)
HLASS4 (uncomfortable around white people)	1.8 (0.9)
HLASS5 (shame)	1.9 (1.0)
HLASS6 (a loss of concentration)	1.7 (0.9)
HLASS7 (feel isolated or distant from other people)	1.7 (0.9)
HLASS8 (a loss of sleep)	1.5 (0.8)
HLASS9 (rage)	1.6 (1.0)
HLASS10 (fearful or distrust the intention of white people)	2.1 (1.1)
HLASS11 (feel like it is happening again)	2.3 (1.1)
HLASS12 (feel like avoiding places or people that remind you of these losses)	1.8 (1.0)
Mean of historical loss associated symptoms scale (HLASS)**	
Mean of depression and anger	2.5 (0.9)
Mean of shame and anxiety	1.7 (0.7)
Mean of re-experiencing, fear, and avoidance	2.1 (0.9)
Mean of hierarchical factor	2.1 (0.7)

*In the table above, the total possible score for the mean of adapted historical loss scale (aHLS) score, cultural loss, intergenerational loss, and distrust and destruction of traditional foods ranged from 1 (never) to 6 (several times a day).

**In the table above, the total possible score for the mean of HLS score, cultural loss, intergenerational loss, and distrust and destruction of traditional foods ranged from 1 (never) to 5 (always).

### 2.4 Procedures and statistical analyses

All procedures of this study were approved by community partners and the University of Hawai‘i Institutional Review Board. Data from this study were based on the Hawaiian Homestead Health Survey administered between 2014 and 2020. The overall comprehensive survey was developed using CBPR approaches and measured demographic variables, general health measures, socio-cultural determinants of health, and psychosocial factors associated with health and health-related behaviors in adult Native Hawaiians residing on Hawaiian homestead lands.

Cognitive interviews were conducted in 2014 to pilot the comprehensive health survey. Cognitive interviewing is a method used to pre-test surveys and gather in-depth responses and insights about items to ensure constructs and surveys are measuring what they intend to measure, and thus, is a form of validity (31). In this case, the research team obtained verbal information about the drafted comprehensive health survey, which included items from the adapted Historical Loss Scale and Historical Loss Associated Symptoms Scale. Cognitive interviews were particularly helpful to reduce response errors and improve comprehension of the overall survey (31).

One survey packet was mailed to each household from select Hawaiian Homestead lands. Survey packets included a consent form, a personalized cover letter with information describing the purpose of the project, and the Hawaiian Homestead Health Survey. Those who consented to participate returned the completed survey in a pre-addressed envelope. Upon receipt of the completed survey, a $25 gift card was provided to the household as compensation for their time. An ID number was assigned to each survey to ensure confidentiality. Survey data were entered in REDCap, a secured, electronic database, then exported to SAS 9.4 to calculate participant characteristics, conduct inter-correlation matrixes, and create Mplus files. Confirmatory Factor Analysis (CFA) and Exploratory Factor Analysis (EFA) were calculated using Mplus Version 8.5.

By default of the MPlus program, CFA consisted of geomin rotated solution, which is a type of oblique rotation, with correlations between factors provided in the Mplus output. Additional specifications were provided within the Mplus program including each item being treated as a categorical variable. For the purpose of this study, standardized factor loadings of the final CFA models are also provided in the figures. Model specifications of EFA analyses included labeling items as categorical variables. Similar to CFA models and by default of the Mplus program, Geomin rotation was utilized for final EFA models. The weighted least square mean and variance adjusted (WLSMV) estimator was utilized for all factor analysis models. This estimator is utilized when data is classified as categorical and uses pairwise deletion. Based on the missing pattern frequencies generated by MPlus, it was confirmed that 463 (out of 491, 94.3%) participants completed the full survey.

In cases where the research team conducted EFA, a cross-validation analysis was utilized. For this process, the final sample was randomly split into two halves, referred to as Sample 1 (*n* = 245) and Sample 2 (*n* = 246). There were no statistically significant differences between demographic variables such as age, gender, number of children, number of people living in household, number of years living on Hawaiian Homestead lands and representation from various homestead communities. During the cross-validation analysis process, Sample 1 was utilized for EFA models, which were then validated through a CFA of the selected EFA model using data from Sample 2. Items were considered to load on a factor if factor loadings were at least 0.4 or greater.

Research over the decades suggest different approaches to reporting goodness-of-fit indices (32). While there are various goodness-of-fit indices that may be considered in structural equation modeling and factor analyses, there are two specific statistics that will be evaluated for the purpose of this study: (1) RMSEA, which considers the error or residual of a model by observing the discrepancy between observed values and predicted values when optimal parameters are chosen and (2) CFI, a goodness of fit statistic that determines the way the structural model fits the observed data adjusting for sample size. In particular, a model was considered to have good or acceptable fit based on goodness of fit statistics, including chi-square, degrees of freedom, Root Mean Square Error of Approximation (RMSEA) and Comparative Fit Index (CFI), with attention primarily given to RMSEA and CFI. For comparative purposes, values are also provided for the Standardized Root Mean Residual (SRMR) and Tucker–Lewis Index (TLI).

RMSEA values range from 0 to 1, with lower values demonstrating better model fit (33, 34). The acceptable cutoff values for the RMSEA are rather debatable. Some argue that RMSEA values of 0.08 are acceptable, while others recommend an RMSEA cutoff value of 0.07 (33). Given the adapted HLS and implementation of new items, the cutoff selected for this study as “acceptable” is an RMSEA of 0.08 or less. SRMR uses similar goodness of fit criteria as RMSEA but differs due to its purpose of measuring differences between the observed correlation and the model implied correlation matrix. In this study, the RMSEA is more highly weighted due to the SRMR being positively biased, with greater bias for studies with smaller N sizes. CFI compares the proposed model to the null model, a model where there is no correlation between all of the observed variables. CFI works well with small samples and tends to be highly correlated with the TLI. CFI also ranges from 0 to 1, however, larger values indicate better model fit. Acceptable model fit is indicated by a CFI value of 0.95 or greater (33).

The minimal cutoff of Cronbach alpha for each scale and construct was set at 0.70. Final models were selected based on a combination of goodness of fit statistics and meaningfulness for the proposed models. Reliability was measured after the final models were selected using Cronbach’s alpha and McDonald’s Omega, which were calculated using JASP software. Different forms of validity include measures such as construct validity, including factorial validity, convergent validity, and discriminant validity (35). Construct validity, a form of validity that indicates that a scale is accurately measuring a construct of interest. Factorial validity exists when an existing hypothesized structure is confirmed through analyses such as CFA. Convergent validity exists when scales or tests overlap in measuring the same construct, while divergent validity exists when scales or tests do not correlate with one another due to the assumption that the scales are measuring two different constructs (35). With these definitions in mind, it is important to note that construct validity, specifically convergent validity, and discriminant validity, may not be achieved in this study due to the primary focus on the aHLS and HLASS. Instead, correlational matrices demonstrating patterns and associations between items and factors of the aHLS and HLASS are reported.

## 3 Results

### 3.1 HLS psychometric models

A summary of the CFA and EFA model results for the aHLS are presented in Tables 5, 6. A Confirmatory Factor Analysis (CFA) was conducted to determine model fit indices for a null model (with 0 correlations set for each item), one-factor model (22), and the suggested 3-factor model proposed by Armenta, Whitbeck, and Habecker (20) based on the items included in the aHLS. The 3-factor CFA model for the purpose of this study and based on the model suggested by Armenta and colleagues (20) comprised cultural loss, cultural mistreatment, and the newly developed items. While the two-factor model with original items (i.e., a two-factor model with the cultural loss and cultural mistreatment items) demonstrated good model fit with an RMSEA of 0.05 and CFI of 1.00, the added items with these two proposed factors demonstrated poor model fit (refer to Table 7).

**Table 5:** Summary of confirmatory factor analyses and exploratory factor analyses results and decision matrix for the adapted historical loss scale (aHLS).

Model	Sample	Chi-square test of model fit	*df*	SRMR	RMSEA	CFI	TLI	Model fit decision
HLS CFA models								
Null	1&2 Combined	9519.83*	28	0.39	0.89	0.00	0.00	Poor fit
One-factor model	1&2 Combined	696.68*	20	0.05	0.29	0.93	0.90	Poor fit
**Adapted 2-factor model (based on the suggested 3-factor model provided by Armenta et al. without the adapted items)** **F1: items 1–3** **F2: items 4 and 6**	**1&2 Combined**	8.345*	**10**	**0.01**	**0.05**	**1.00**	**1.00**	**Excellent fit; acceptable RMSEA, SRMR, and CFI**
Three factor model (based on the suggested model provided by Armenta et al. and the adapted items) F1: items 1–3 F2: items 4 and 6 F3: items 5, 7, and 8	1&2 Combined	388.71*	28	0.04	0.26	0.96	0.93	Acceptable SRMR and CFI, poor RMSEA, poor TLI
Three factor model (based on the suggested model provided by Armenta et al. and the adapted items) F1: items 1–2 F2: items 3, 4, and 6 F3: items 5, 7, and 8	1&2 Combined	578.29*	17	0.05	0.29	0.94	0.90	Acceptable SRMR, poor RMSEA, CFI, TLI

In the table above, the *indicates a chi-square test of model fit with a *p*-value that is less than 0.05 indicating statistical significance. The bolded row indicates the final model selected for this study.

**Table 6:** Summary of confirmatory factor analyses and exploratory factor analyses results and decision matrix for the adapted historical loss scale (aHLS).

Model	Sample	Chi-square test of model fit	*df*	SRMR	RMSEA	CFI	TLI	Model fit decision
HLS cross-validation models (based on 8-items)								
One-factor EFA model (items 1–8)	Sample 1	450.77*	20	0.09	0.30	0.93	0.90	Poor fit
Two-factor EFA model (items 1–8)	Sample 1	167.84*	13	0.04	0.23	0.98	0.95	Acceptable SRMR, CFI, and TLI, poor RMSEA
Three-factor EFA model (items 1–8)	Sample 1	87.51*	7	0.03	0.22	0.99	0.95	Acceptable SRMR, CFI, and TLI, poor RMSEA
Four-factor EFA model* (items 1–8) F1: items 1–3 F2: items 4–5 F3: items 6–7 F4: item 8	**Sample 1**	2.46*	**2**	**0.01**	**0.03**	**1.00**	**1.00**	**Excellent fit; acceptable RMSEA, SRMR, and CFI**
HLS cross-validation methods (based on 7-items)								
One-factor EFA model (items 1–7)	Sample 1	368.11*	21	0.10	0.33	0.94	0.91	Poor fit
Two-factor EFA model (items 1–7)	Sample 1	107.04*	21	0.04	0.23	0.98	0.96	Acceptable SRMR, CFI, and TLI, poor RMSEA
**Three-factor EFA model (items 1–7)** F1: items 1–3 F2: items 4–5 F3: items 6–7	**Sample 1**	5.65*	**21**	**0.01**	**0.06**	**1.00**	**1.00**	**Excellent fit; acceptable RMSEA, SRMR, and CFI**
CFA of 3-factor EFA model F1: items 1–3 F2: items 4–5 F3: items 6–7	Sample 2	31.67	21	0.01	0.09	1.00	0.99	Good fit
Hierarchical CFA of 3-factor EFA model F1: items 1–3 F2: items 4–5 F3: items 6–7 F4: F1 F2 F3	Sample 2	31.67	21	0.01	0.09	1.00	0.99	Good fit

In the table above, the *indicates a chi-square test of model fit with a *p*-value that is less than 0.05 indicating statistical significance. The bolded row indicates the final model selected for this study.

**Table 7:** Factor structure loadings for confirmatory factor analysis based on Armenta’s model.

Item no.	Adapted historical loss scale (aHLS)	Factor 1: cultural loss	Factor 2: cultural mistreatment
Confirmatory factor analysis based on Armenta’s model (Geomin model specification, standardized factor loadings, items 1–4 and 6, both samples, *n* = 491)			
1	The taking of our land	0.827	
2	Fewer and fewer people using our traditional language	0.882	
3	Destruction of our culture and traditional spiritual ways	0.925	
4	Loss of respect for elders by our children and grandchildren		0.718
6	Distrust, resentment, or fear toward white people		0.726

**Figure 1:**
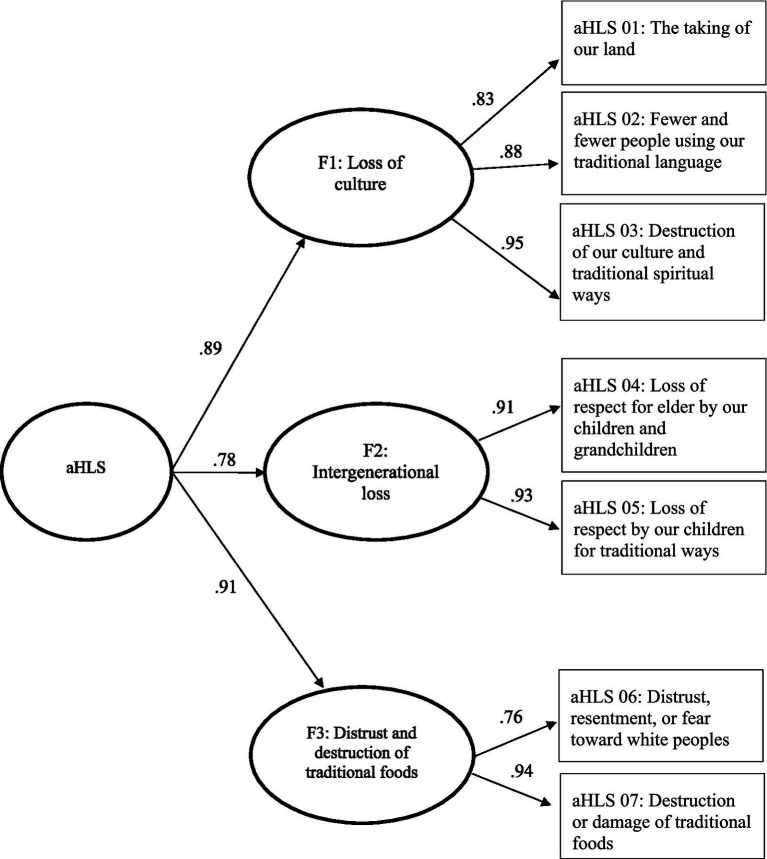
Psychometric model of aHLS as a hierarchical 3-factor model with standardized factor loadings.

Due to poor model fit for the CFA models, an Exploratory Factor Analysis (EFA) was conducted using a cross-validation method. The EFA sample matrix correlation yielded the following eigenvalues based on data from Sample 1: 5.628, 0.734, 0.478, 0.462, 0.230, 0.188, 0.169, and 0.112. Thus, eigenvalues indicated that factor 1 represents the greatest magnitude of importance for the EFA models. However, based on a combination of model fit indices and meaningfulness of factors for sample 1, the 7 item, 3-factor model was selected as the best EFA model with an RMSEA of 0.06 and a CFI of 1.00. Of the eight adapted items, one item (Item 8) was omitted as it operated as a one-item factor when included in the EFA (refer to Table 8). A CFA of the selected 7-item, 3-factor model continued to demonstrate good fit based on data from Sample 2 with an RMSEA of 0.08 and CFI of 1.00. Figure 1 visually depicts the final selected psychometric model of aHLS as a hierarchical 3-factor model with standardized factor loadings.

**Table 8:** Factor structure loadings of final exploratory and confirmatory factor analysis models for the adapted historical loss scale (aHLS).

Item no.	Adapted historical loss scale (aHLS)	Factor 1: loss of culture	Factor 2: inter-generational loss	Factor 3: distrust and destruction of traditional foods	Factor 4: destruction of natural resources
Exploratory factor analysis 4-factor model (Geomin rotated model results, all items, sample 1, *n* = 245)					
1	The taking of our land	0.618*	−0.081*	0.054	0.324
2	Fewer and fewer people using our traditional language	0.772*	0.036	0.128	−0.044
3	Destruction of our culture and traditional spiritual ways	0.909*	0.089	−0.084	0.031
4	Loss of respect for elders by our children and grandchildren	−0.012	0.971*	−0.014	0.022
5	Loss of respect by our children for traditional ways	0.064	0.808*	0.094	0.006
6	Distrust, resentment, or fear toward white people	−0.011	0.041	0.925*	0.005
7	Destruction or damage of traditional foods	0.058	−0.002	0.608*	0.284*
8	The destruction of natural resources and beauty due to pollution, mining, and other industries	0.002	0.083	−0.001	0.975*

Item no.	Adapted historical loss scale (aHLS)	Factor 1: loss of culture	Factor 2: inter-generational loss	Factor 3: distrust and destruction of traditional foods
Exploratory factor analysis 3-factor model (Geomin rotated model results, items 1–7, sample 1, *n* = 245)				
1	The taking of our land	0.597*	−0.028	0.287*
2	Fewer and fewer people using our traditional language	0.653*	0.014	0.236*
3	Destruction of our culture and traditional spiritual ways	0.910*	0.054	−0.003
4	Loss of respect for elders by our children and grandchildren	0.188	0.746*	−0.008
5	Loss of respect by our children for traditional ways	−0.003	0.972*	0.059
6	Distrust, resentment, or fear toward white peoples	0.000	0.016	0.877*
7	Destruction or damage of traditional foods	0.008	0.005	0.860*

Item no.	Adapted historical loss scale (aHLS)	Factor 1: loss of culture	Factor 2: inter-generational loss	Factor 3: distrust and destruction of traditional foods
Final confirmatory factor analysis of 3-factor exploratory factor model (Geomin model specification, standardized factor loadings, items 1–7, sample 2, *n* = 246)				
1	The taking of our land	**0.831**		
2	Fewer and fewer people using our traditional language	**0.884**		
3	Destruction of our culture and traditional spiritual ways	**0.949**		
4	Loss of respect for elders by our children and grandchildren		**0.906**	
5	Loss of respect by our children for traditional ways		**0.931**	
6	Distrust, resentment, or fear toward white peoples			**0.759**
7	Destruction or damage of traditional foods			**0.936**

In the table above, the *indicates significance at 0.05 level. Loadings greater than 0.4 are bolded.

### 3.2 HLASS psychometric models

A summary of the CFA and EFA model results for the HLASS are presented in Table 9. Goodness of fit statistics were assessed for the HLASS using CFA and EFA models. CFA models were conducted to determine goodness of fit statistics for a null model (with 0 correlations set for each item), one-factor model (27), and the suggested 2-factor model proposed by Whitbeck and colleagues (22). The suggested 2-factor model comprised anxiety/depression and anger/avoidance. Based on poor model fit for the HLASS CFA models, an EFA was conducted using a cross-validation method.

**Table 9:** Summary of confirmatory factor analyses and exploratory factor analyses results and decision matrix for the historical loss associated symptoms scale (HLASS).

Model	Sample	Chi-square test of model fit	*df*	SRMR	RMSEA	CFI	TLI	Model fit decision
**HLASS CFA models**								
Null	1&2 Combined	7283.10*	66	0.36	0.51	0.00	0.00	Poor fit
One-factor model	1&2 Combined	766.10*	66	0.07	0.17	0.91	0.90	Poor fit
Two-factor model F1: items 1, 3, 6, 7, and 8 F2: items 2, 4, 5, 9, 10, 11, 12	1&2 Combined	679.92*	53	0.07	0.16	0.92	0.91	Poor fit
**HLASS EFA models (12 items)**								
One-factor EFA model	Sample 1	496.55*	54	0.12	0.19	0.90	0.88	Poor fit
Two-factor EFA model	Sample 1	238.94*	43	0.07	0.14	0.96	0.93	Acceptable SRMR and CFI, poor RMSEA and TLI
**Three-factor EFA model** **items 1, 2** **items 5, 6, 7, 8,** **items 10, 11, 12**	**Sample 1**	70.06*	**33**	**0.03**	**0.07**	**0.99**	**0.98**	**Good fit; acceptable RMSEA, SRMR, CFI, and TLI**
Four-factor EFA model	Sample 1	No Convergence	n/a	n/a	n/a	n/a	n/a	No Convergence
Five-factor EFA model	Sample 1	31.67*	16	0.02	0.06	1.00	0.99	**Excellent fit; acceptable RMSEA, SRMR, CFI, and TLI**
Six-factor EFA model	Sample 1	No convergence	n/a	n/a	n/a	n/a	n/a	No convergence
**HLASS EFA models (9 items after dropping items 3, 4, and 9)**								
One-factor EFA model	Sample 1	406.88*	27	0.15	0.24	0.89	0.86	Poor fit
Two-factor EFA model	Sample 1	175.57*	19	0.07	0.19	0.96	0.92	Acceptable SRMR and CFI, poor RMSEA and TLI
**Three-factor EFA model** **items 1, 2** **items 5, 6, 7, 8,** **items 10, 11, 12**	**Sample 1**	28.44*	**12**	**0.02**	**0.07**	**1.00**	**0.99**	**Good fit; acceptable RMSEA, SRMR, CFI, and TLI**
**HLASS CFA of EFA model**								
CFA of the 3-factor EFA model F1: items 1, 2 F2: items 5, 6, 7, 8 F3: items 10, 11, 12	Sample 2	61.24*	24	0.03	0.08	0.99	0.98	**Acceptable fit; acceptable RMSEA, SRMR, CFI, and TLI**
Hierarchical CFA of the 3-factor EFA model F1: items 1, 2 F2: items 5, 6, 7, 8 F3: items 10, 11, 12 F4: F1 F2 F3	Sample 2	61.24*	24	0.03	0.08	0.99	0.98	**Acceptable fit; acceptable RMSEA, SRMR, CFI, and TLI**

In the table above, the *indicates a chi-square test of model fit with a *p*-value that is less than 0.05 indicating statistical significance. The bolded row indicates the final model selected for this study.

**Figure 2:**
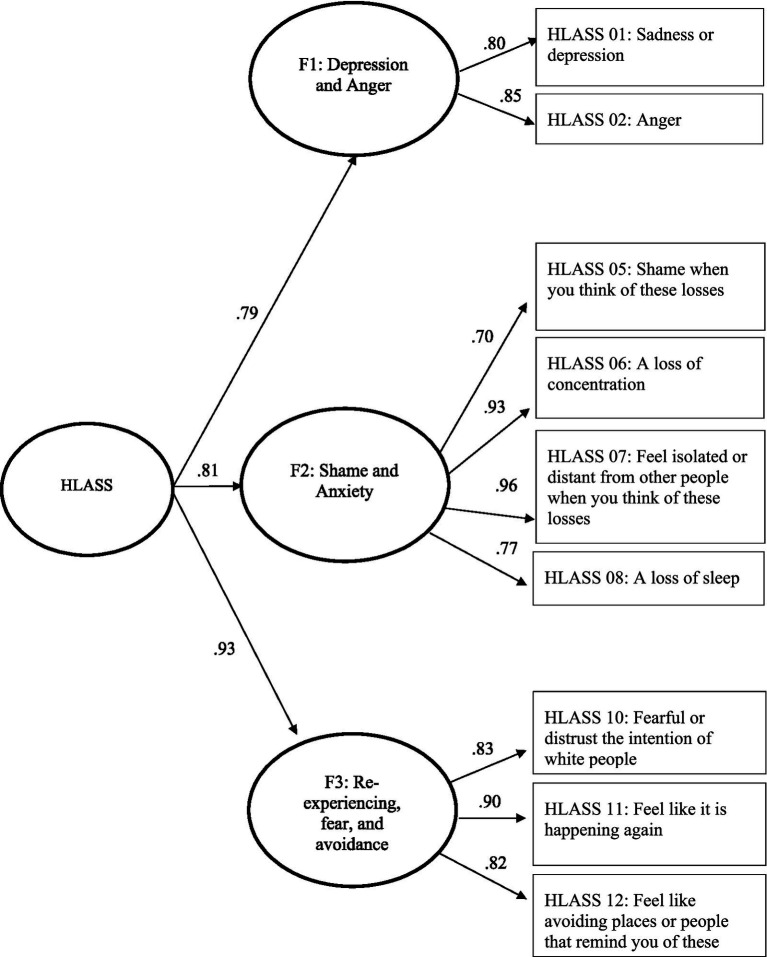
Psychometric model of HLASS as a hierarchical 3-factor model with standardized factor loadings.

The EFA sample matrix correlation yielded the following eigenvalues based on data from Sample 1: 6.744, 1.386, 0.970, 0.621, 0.515, 0.461, 0.347, 0.295, 0.276, 0.173, 0.129, and 0.083. Thus, eigenvalues supported a multidimensional model for the HLASS, with 2 or 3 factors demonstrating magnitude of importance for the EFA models. Based on goodness of fit statistics for the EFA models using data from Sample 1, the three-factor model was selected. Goodness of fit statistics for the 3-factor EFA model with all items were acceptable with an RMSEA of 0.07 and a CFI of 0.99. However, of the 12 items included in the HLASS, three items were omitted. Two of the 12 items did not load on a factor including HLASS Item 4: Uncomfortable around white people (when you think of these losses) and HLASS Item 9: Rage, whereas Item 3: Anxiety or nervousness double-loaded on Factor 1 and 2 (refer to Table 10). Figure 2 visually depicts the final selected psychometric model of HLASS as a hierarchical 3-factor model with standardized factor loadings.

**Table 10:** Factor structure loadings of final exploratory and confirmatory factor analysis models for the historical loss associated symptoms scale (HLASS).

Item no.	Historical loss associated symptoms scale (HLASS)	Factor 1: depression and anger	Factor 2: shame and anxiety	Factor 3: re-experiencing, fear, and avoidance
Exploratory factor analysis 3-factor model (Geomin rotated model results, all items, sample 1, *n* = 245)				
1	Sadness or depression	0.734*	0.045	0.089
2	Anger	0.751*	−0.030	0.194
3	Anxiety or nervousness	0.539*	0.469*	−0.014
4	Uncomfortable around white people (when you think of these losses)	0.156	0.375*	0.388*
5	Shame (when you think of these losses)	0.216*	**0.556**	0.046
6	A loss of concentration	−0.009	**0.930**	0.036
7	Feel isolated or distant from other people (when you think of these losses)	−0.008	0.756*	0.250*
8	A loss of sleep	0.137	0.742*	−0.022
9	Rage	0.318*	0.223*	0.360*
10	Fearful or distrust the intention of white people	−0.053	0.013	0.979*
11	Feel like it is happening again	0.081	−0.042	0.874*
12	Feel like avoiding places or people that remind you of these losses	−0.002	0.225*	0.624*

Item no.	Historical loss associated symptoms scale (HLASS)	Factor 1: depression and anger	Factor 2: shame and anxiety	Factor 3: re-experiencing, fear, and avoidance
Exploratory factor analysis 3-factor model (Geomin rotated model results, items 1–2, 5–8, 10–12, sample 1, *n* = 245)				
1	Sadness or depression	0.731*	0.061	0.009
2	Anger	0.907*	−0.007	−0.005
5	Shame (when you think of these losses)	0.225*	0.561*	0.013
6	A loss of concentration	−0.026	0.950*	0.008
7	Feel isolated or distant from other people (when you think of these losses)	−0.005	0.781*	0.221*
8	A loss of sleep	0.094	0.778*	−0.054
10	Fearful or distrust the intention of white people	−0.023	0.082	0.895*
11	Feel like it is happening again	0.124	−0.012	0.859*
12	Feel like avoiding places or people that remind you of these losses	0.018	0.258*	0.595*

Item no.	Historical loss associated symptoms scale (HLASS)	Factor 1: depression and anger	Factor 2: shame and anxiety	Factor 3: re-experiencing, fear, and avoidance
Final confirmatory factor analysis of 3-factor exploratory factor model (Geomin model specification, standardized factor loadings, sample 2, *n* = 246)				
1	Sadness or depression	0.799		
2	Anger	0.850		
5	Shame (when you think of these losses)		0.702	
6	A loss of concentration		0.927	
7	Feel isolated or distant from other people (when you think of these losses)		0.955	
8	A loss of sleep		0.772	
10	Fearful or distrust the intention of white people			0.830
11	Feel like it is happening again			0.896
12	Feel like avoiding places or people that remind you of these losses			0.815

In the table above, the *indicates significance at 0.05 level. Loadings greater than 0.4 are bolded.

### 3.3 Reliability and inter-correlation matrix

Tables 11, 12 present the internal consistency coefficients (i.e., reliability) for the aHLS and HLASS scales and suggested subscales. Overall, the results demonstrated good reliability based on McDonald’s Omega and the standardized Cronbach alpha, which ranged from 0.76 to 0.92. Inter-correlation matrices were generated to determine the bivariate relationships among the items and final factors included in the aHLS and HLASS. As shown in Tables 11–13, all the inter-factor and inter-scale correlations were statistically significant at the *p* < 0.01 level. The correlations demonstrated positive and significant relationships between the set of aHLS and HLASS items and factors, with correlations for the aHLS ranging from 0.39 to 0.93; correlations for the HLASS ranging from 0.25 to 0.90; and the correlation between aHLS and HLASS being 0.59. The inter-correlation matrices also demonstrated higher correlations for respective constructs.

**Table 11:** Reliability and inter-correlation matrix of the adapted historical loss scale (aHLS).

	Omega^a^	Alpha^a^	1	2	3	4	5	6	7	8	9	10	11	12	13
1. HLS item 1 (the taking of land)	–	–													
2. HLS item 2 (fewer and fewer people using our traditional language)	–	–	0.70												
3. HLS item 3 (destruction of our culture and traditional spiritual ways)	–	–	0.73	0.79											
4. HLS item 4 (loss of respect for elders by our children and grandchildren)	–	–	0.48	0.58	0.59										
5. HLS item 5 (loss of respect by our children for traditional ways)	–	–	0.50	0.58	0.65	0.83									
6. HLS item 6 (distrust, resentment, or fear toward white peoples)	–	–	0.55	0.56	0.58	0.47	0.52								
7. HLS item 7 (destruction or damage of traditional foods)	–	–	0.61	0.61	0.68	0.51	0.58	0.69							
8. EFA factor 01: cultural loss	0.90	0.89	0.89	0.91	0.93	0.61	0.64	0.62	0.70						
9. EFA factor 02: intergenerational loss	0.64	0.64	0.60	0.66	0.68	0.86	0.80	0.85	0.71	0.71					
10. EFA factor 01: cultural loss mean score	0.90	0.89	0.89	0.91	0.93	0.61	0.64	0.62	0.70	1.00	0.71				
11. EFA factor 02: intergenerational loss mean score	0.91	0.91	0.39	0.61	0.65	0.96	0.96	0.52	0.57	0.65	0.87	0.65			
12. EFA factor 03: distrust and destruction of traditional foods mean score	0.82	0.82	0.64	0.64	0.68	0.53	0.59	0.92	0.92	0.71	0.85	0.71	0.59		
13. HLS hierarchical mean score	0.92	0.91	0.80	0.84	0.88	0.78	0.82	0.78	0.83	0.89	0.93	0.89	0.88	0.88	

All inter-factor and inter-scale correlations were statistically significant at the *p* < 0.01 level.

^a^McDonald’s omega and standardized Cronbach’s alpha were based on the seven items of the HLS. Correlations were based on the mean of the three factors and the hierarchical factor of the HLS three-factor EFA model (items 1–7).

**Table 12:** Reliability and inter-correlation matrix of the historical loss associated symptoms scale (HLASS).

	Omega^a^	Alpha^a^	1	2	3	4	5	6	7	8	9	10	11	12
1. HLASS item 1 (sadness or depression)	–	–												
2. HLASS item 2 (anger)	–	–	0.61											
3. HLASS item 5 (shame when you think of these losses)	–	–	0.30	0.34										
4. HLASS item 6 (A loss of concentration)	–	–	0.36	0.35	0.52									
5. HLASS item 7 (feel isolated or distant from other people when you think of these losses)	–	–	0.34	0.39	0.53	0.76								
6. HLASS item 8 (A loss of sleep)	–	–	0.34	0.28	0.37	0.62	0.59							
7. HLASS item 10 (fearful or distrust the intention of white people)	–	–	0.35	0.45	0.29	0.43	0.47	0.30						
8. HLASS item 11 (feel like it is happening again)	–	–	0.45	0.50	0.38	0.43	0.47	0.25	0.75					
9. HLASS item 12 (feel like avoiding places or people that remind you of these losses)	–	–	0.34	0.38	0.36	0.41	0.50	0.38	0.57	0.58				
10. Factor 01: depression and anger mean score	0.75	0.75	0.90	0.90	0.35	0.39	0.41	0.35	0.45	0.53	0.40			
11. Factor 02: shame and anxiety mean score	0.84	0.83	0.41	0.42	0.76	0.88	0.88	0.77	0.46	0.48	0.51	0.46		
12. Factor 03: re-experiencing, fear, and avoidance mean score	0.85	0.84	0.44	0.52	0.40	0.49	0.56	0.35	0.89	0.90	0.81	0.53	0.55	
13. HLASS Hierarchical mean score	0.76	76	0.73	0.77	0.59	0.69	0.72	0.57	0.74	0.79	0.70	0.83	0.78	0.86

All inter-factor and inter-scale correlations were statistically significant at the *p* < 0.01 level.

^a^McDonald’s omega and standardized Cronbach’s alpha were based on the selected 10 items from the HLASS. Correlations were based on the mean of the three factors and the hierarchical factor of the HLASS.

**Table 13:** Reliability and inter-correlation matrix of the adapted historical loss scale (aHLS) and the historical loss associated symptoms scale (HLASS).

	1	2	3	4	5	6	7	8
1. HLS F1: cultural loss mean score								
2. HLS F2: intergenerational loss mean score	0.65							
3. HLS F3: distrust and destruction of traditional foods mean score	0.71	0.59						
4. HLS hierarchical mean score	0.89	0.86	0.88					
5. HLASS F1: depression and anger mean score	0.46	0.41	0.42	0.50				
6. HLASS F2: shame and anxiety mean score	0.35	0.30	0.40	0.40	0.46			
7. HLASS F3: re-experiencing, fear, and avoidance mean score	0.50	0.38	0.55	0.54	0.53	0.55		
8. HLASS hierarchical mean score	0.54	0.45	0.56	0.59	0.83	0.78	0.86	

All inter-factor and inter-scale correlations were statistically significant at the *p* < 0.01 level.

## 4 Discussion

The overall purpose of this study was to estimate psychometric properties of the aHLS and HLASS in a Native Hawaiian sample. The findings of this study suggested both the aHLS and HLASS may be most acceptable as a three-factor model when measuring Historical Loss and associated symptoms in Native Hawaiian communities. The aHLS comprised factors that assessed for loss of culture, intergenerational loss, and distrust and destruction of traditional foods. The HLASS comprised factors that measured depression and anger, shame and anxiety, and re-experiencing, fear, and avoidance. The aHLS, which used seven of the eight adapted items proposed by community members and leaders, demonstrated good model fit for the Native Hawaiian sample. The proposed 3-factor model of the HLASS overlapped with the emotional responses identified through the original factor structure proposed by Whitbeck and colleagues (22). The results of this study also demonstrated good reliability based on McDonald’s Omega and the standardized Cronbach alpha.

The HLS and HLASS were originally developed as standardized measures to assess the direct and indirect impacts of Historical Loss on other Indigenous populations, specifically North American Indigenous communities (22). Our findings support the use of the aHLS and HLASS in Native Hawaiian populations. Our findings further validate the importance of measuring Historical Loss and associated symptoms among Native Hawaiians as multidimensional constructs. This aligns with other research that supports the importance of addressing trauma, including cultural and historical trauma and community injustice, through a multidimensional lens (19, 36). For instance, the aHLS as a 3-factor model was most highly correlated with depression and anger followed by shame and anxiety. These associated symptoms of Historical Loss have substantial public health impacts and are negatively associated with multi-level social determinants of health, quality of life, as well as overall health including increased risk of chronic health conditions (37–39). By addressing Historical Loss as historical trauma, and thus, a multidimensional determinant of health, cultural strengths and values that address Historical Loss may directly and indirectly increase the resiliency of Native Hawaiians; thereby fostering wellness and wellbeing.

The development of the aHLS and pilot testing of the HLASS through community consultation helped to increase the face validity of these constructs. This is not surprising given the ongoing research that emphasizes the importance of taking a community-based and culturally sensitive approach to research with Native and Indigenous communities at large (1, 40, 41). In this case, engaging community in every step of the research process aided in the adaptation of culturally sensitive and appropriate measures of Historical Loss and associated symptoms of Historical Loss. The aHLS and the HLASS can be used to reliably assess the frequency with which Native Hawaiians think of Historical Loss and the negative emotions that are experienced when Native Hawaiians think of these Historical Loss.

Frequency distributions and mean scores indicated that on average, participants would think of the historical loss items on a monthly basis, with participants indicating greatest frequency of thinking about the loss of respect for elders by our children and grandchildren and the destruction of natural resources and beauty due to pollution, mining, and other industries. These findings correspond with Whitbeck et al.’s original published article on HLS, which indicated that the majority of participants would think of cultural loss or cultural mistreatment items (i.e., loss of our land, the loss of our family ties because of boarding schools, the loss of families from the reservation to government relocation, the loss of self-respect from poor treatment by government officials, and the loss of trust in White individuals from broken treaties) “never” or “yearly” during special times of the year. On the other hand, items from Whitbeck et al.’s original scale that assessed for loss of language, loss of traditional spiritual ways, loss of culture, loss of people from the effects of alcoholism, loss of respect by our children and grandchildren for elders, loss of our people through early death, and loss of respect by children tended to have higher frequencies, with participants reporting thinking about these losses on a daily basis.

Additionally, reported associated symptoms were seldom experienced when thinking about the historical loss items. It is possible these lower mean scores are attributed to the final items that were ultimately adapted and included in these surveys. On the other hand, it is possible participants from this study do not necessarily view these items as “loss” or “historical loss” due to the ongoing resistance and resilience that continues to preserve and perpetuate Hawaiian knowledge, intergenerational strengths, and ways of knowing. This was showcased during our initial meetings, where community leaders and partners indicated the need to change items such as “loss of our land” to the “taking of our land.” Re-framing this item demonstrates the way in which land is still present and nourishing us as people, but the current political and economic power structures limit the ability to be intimate with land due to the taking of land. This also sheds light on the ongoing fight for justice and self-determination, with implications for historical, present, and future loss. Thus, future studies that aims to explore historical trauma must also consider the impacts of larger and systemic barriers that also play a role in the health and wellbeing of Native Hawaiians, which extends further to Indigenous Peoples at large.

Overall, an exploration of historical trauma and symptoms associated with historical loss and historical trauma continue to be an urgent and important public health topic, especially given the social, cultural, economic, and political implications of present-day adversities. These adversities range to include the exacerbation of health factors through large public health issues including the COVID-19 pandemic (42), increased crises related to housing and affordable living, and devasting impacts of the natural environment, including the wildfires of Maui. Gaining a better understanding of Historical Loss and historical loss symptoms may assist with more upstream, systemic solutions that may better the health and wellbeing of Native Hawaiian through a social justice lens. For example, by associating intergenerational loss with current mental and emotional health conditions of Native Hawaiians, community, researchers, academicians, and practitioners will be able to illustrate the severity of addressing colonial atrocities like water rights issues in Hawaiʻi. To illustrate, historical and contemporary American capitalist ventures in Hawaiʻi, particularly plantations and tourism, resulted in the diversion of natural water ways, impacting the ancestral ways of life in which Native Hawaiians interact with land. Natural resources and agricultural systems that maintained Native Hawaiian health have been threatened by desecration, serving as a catalyst of health inequities for contemporary Native Hawaiians and the increased reliance on imported foods. Applying findings from aHLS and HLASS quantify the need to restore ancestral ways of life, including natural flowing waters, as means to address intergenerational loss.

### 4.1 Limitations

The findings from this study are based on cross-sectional survey data. As with any cross-sectional study, the findings from this study are limited to data based on one point in time. As a result, causal statements may not be drawn. Furthermore, responses may have differed depending on the date in which the survey was administered. Survey administration took place between 2014 and 2020, with various significant and historical events taking place during this time, specifically in the context of Hawaiian history. For example, in 2015 and 2019, the threat of a Thirty Meter Telescope atop a sacred mountain, Mauna Kea, caused an uprising among many Native Hawaiians. Such an event illustrates the continual contention between Native Hawaiians and settlers who perpetuate Native Hawaiian cultural erasure, degradation, and trauma. These contextual factors are therefore important to consider for data interpretation. For instance, the forementioned present-day historical trauma may intersect with health consequences that stem from historical trauma of past, thereby resulting in negative health outcomes, including the inequities experienced by contemporary Native Hawaiians. Furthermore, although the data included participants from various Hawaiian Homestead communities, these findings may not be generalizable to other Hawaiians, including those who currently reside or do not reside on Hawaiian Homestead Lands as well as those who currently reside or do not reside in Hawai‘i. Therefore, future research should consider further psychometric analysis of the proposed aHLS and HLASS among Hawaiian communities at large.

### 4.2 Conclusion

Measurement of the aHLS and HLASS in the Native Hawaiian homestead population paves the way for quantitative assessments of historical trauma in Native Hawaiian communities at large. A better understanding of how the three factors identified in the aHLS relate to the three factors identified in the HLASS is needed to better understand the relationship between historical loss and associated symptoms. For instance, future studies could consider if thoughts related to the loss of culture, intergenerational loss, destruction of traditional foods, and distrust illicit the same reactions as measured by the HLASS. Additionally, future research should examine the strength of association between thoughts of certain losses and one HLASS factor compared to the others. This may also have implications for future research and practice that explores historical loss; symptoms associated with thoughts that influence mental, physical, and spiritual health in Native Hawaiian and Indigenous populations; and potential mediators and moderators of these relationships including impersonal or structural racism, cultural identity, cultural practice, or demographics such as age.

Most importantly, findings from this study may pave the way for efforts that aim to heal historical loss and associated symptoms among the Native Hawaiian community. An increased understanding of historical loss and its impact on health and wellbeing betters our understanding of resistance, resiliency, and the ability to overcome historical loss as a deep-seated determinant of health that stems from cultural and historical trauma and oppression. Initiatives that aim to heal the impact of listorical loss, including restoration of Native Hawaiian ways of knowing and cultural practices, including ‘Āina connectedness and land back, are critical in fostering mauli ola, optimal health and wellbeing, for the Native Hawaiian community and Indigenous Peoples at large.

## Data availability statement

The datasets presented in this article are not readily available because the data included in this project belongs to community partners. To increase data sovereignty, datasets are not provided publicly. Requests to access the datasets should be directed to MA, antoniom@hawaii.edu.

## Ethics statement

The studies involving humans were approved by University of Hawai‘i at Mānoa Institutional Review Board. The studies were conducted in accordance with the local legislation and institutional requirements. The ethics committee/institutional review board waived the requirement of written informed consent for participation from the participants or the participants’ legal guardians/next of kin because of increased anonymity. Instead, survey packets were mailed to each household from select Hawaiian Homestead lands. Those who consented to participate returned the completed survey in a pre-addressed envelope. Completing the survey warranted consent.

## Author contributions

MA: Conceptualization, Data Curation, Formal Analysis, Funding Acquisition, Methodology, Software, Validation, Visualization, Writing – original draft, Writing – review & editing. SK: Conceptualization, Formal Analysis, Methodology, Software, Validation, Writing – original draft, Writing – review & editing. CI: Conceptualization, Funding Acquisition, Methodology, Validation, Writing – original draft, Writing – review & editing. MW: Writing – original draft, Writing – review & editing. AD: Conceptualization, Funding Acquisition, Methodology, Project administration, Validation, Writing – original draft, Writing – review & editing. BK: Conceptualization, Funding Acquisition, Methodology, Project administration, Validation, Writing – original draft, Writing – review & editing. MKe: Conceptualization, Funding Acquisition, Methodology, Project administration, Validation, Writing – original draft, Writing – review & editing. SM: Writing – original draft, Writing – review & editing. KC: Conceptualization, Funding Acquisition, Methodology, Validation, Writing – original draft, Writing – review & editing. SA: Methodology, Validation, Writing – original draft, Writing – review & editing. MKa: Methodology, Validation, Writing – original draft, Writing – review & editing. JK: Conceptualization, Methodology, Funding Acquisition, Supervision, Validation, Writing – original draft, Writing – review & editing.
